# Stabilizers influence drug–polymer interactions and physicochemical properties of disulfiram-loaded poly-lactide-co-glycolide nanoparticles

**DOI:** 10.4155/fsoa-2017-0091

**Published:** 2017-12-13

**Authors:** Muddasarul Hoda, Shamim Akhtar Sufi, Bindumadhuri Cavuturu, Rukkumani Rajagopalan

**Affiliations:** 1 Department of Biological Sciences, Aliah University, Kolkata, 700 156, India; 2 Department of Biochemistry & Molecular Biology, School of Life Sciences, Pondicherry University, Puducherry, 605 014, India; 3 Interdisciplinary Programme for Life Sciences, Pondicherry University, Puducherry, 605 014, India; 4 Department of Biotechnology, School of Life Sciences, Pondicherry University, Puducherry, 605 014, India

**Keywords:** drug delivery, drug–polymer interaction, nanoparticles, PLGA, polymer degradation, stabilizers

## Abstract

**Aim:**

Stabilizers are known to be an integral component of polymeric nanostructures. Ideally, they manipulate physicochemical properties of nanoparticles. Based on this hypothesis, we demonstrated that disulfiram (drug) and Poly-lactide-co-glycolide (polymer) interactions and physicochemical properties of their nanoparticles formulations are significantly influenced by the choice of stabilizers.

**Methodology:**

Electron microscopy, differential scanning calorimetry, x-ray diffraction, Raman spectrum analysis, isothermal titration calorimetry and *in silico* docking studies were performed.

**Results & discussion:**

Polysorbate 80 imparted highest crystallinity while Triton-X 100 imparted highest rigidity, possibly influencing drug bioavailability, blood-retention time, cellular uptake and sustained drug release. All the molecular interactions were hydrophobic in nature and entropy driven. Therefore, polymeric nanoparticles may be critically manipulated to streamline the passive targeting of drug-loaded nanoparticles.

## Lay abstract

Polymeric nanoparticles are futuristic drug-delivering platforms that have many potential advantages above conventional drug-delivery tools. They are mainly composed of a polymer, stabilizer and the therapeutic ingredient. A number of researches are on-going to improvise various characteristics of polymeric nanoparticles, in order to enhance its efficacy. The current study is one such domain where we emphasize on identifying potential stabilizing factors that are involved in nanoparticles formation and their drug entrapment and release properties.

Nanoparticles (NPs) are among the futuristic drug delivery devices. They are potentially involved in active and passive targeting of drugs and active pharmaceutical ingredients. Ideally, they minimize the adverse effects and enhance localized distribution of the therapeutic molecules. They have the potential to release the drugs for extended period of time, thus altering pharmacokinetics of the drug, resulting in lowered dosage regimens [1]. The hallmark of nanoparticles as an ideal drug-delivery vehicle are based on characteristics such as toxicology, interaction with the drug under study, drug-loading ability, sustained drug-release patterns, blood-residence time (BRT), cellular uptake, biodegradation and elimination [2]. Polymeric biodegradable nanoparticles are among many such potential drug delivery vehicles. Polymers such as Poly-lactide-co-glycolide (PLGA), poly-lactic acid and poly-caprolactam have been extensively studied. PLGA, in principle, degrades into lactic acid and glycolic acid, which are eliminated through the general metabolic pathways of the biological system [3]. It has also been reported to have good drug-entrapment ability, and is a promising delivery vehicle for hydrophobic drugs which otherwise fail to enter the target system because of extremely limited bioavailability.

Stabilizers are one of the important components of polymeric nanoparticles. They directly influence the drug payload of nanoparticles [4]. For a specific drug and polymer combination, different stabilizers may induce different drug-loading capacities in the polymeric nanoparticles, in spite of being of more or less similar particle size [5]. In other words, stabilizers may directly affect the drug payload of NPs without significantly affecting the NPs’ size. The drug-release patterns and other physicochemical characteristics of nanoparticles may also be directly influenced by stabilizers. There are reports focusing on drug–polymer interactions with specific drug or polymer as [6]. There are also reports on the modification of individual stabilizers to study of the effect of specific functional groups in the development of stable polymeric NPs [7]. However, extensive researches are needed to understand the significance of various stabilizers in synthesis of NPs with tailor-made properties.

Disulfiram (DSF), an aldehyde dehyrogenase inhibitor and a potential anticancer drug has been of late studied as a model for drug entrapment studies in PLGA nanoparticles due to its hydrophobic nature and limited bioavailability [8]. DSF-loaded PLGA NPs (DNPs) has previously been synthesized in our lab using three different stabilizers, viz a viz. polysorbate 80 (PS80), Triton-X 100^®^ (T100), and Pluronic 188^®^ (P188) [5]. Though, all the NPs looked morphologically similar, they potentially differed in their physicochemical properties. In our previous study, we had established that these differentially stabilized NPs were capable of changing the pharmacokinetics of the drug, along with drug-loading capacity [5].

In the present study, we further explore the influence of various stabilizers on physicochemical characterization of PLGA NPs, with special reference to drug–polymer and polymer–stabilizer interactions. We also hypothesized that NP synthesis is a thermodynamically driven process, and thermodynamic studies of NP synthesis, was probably studied for the first time using isothermal titration calorimetry (ITC).

## Materials & methods

### Chemicals

PLGA (75:25), DSF and Pluronic 188^®^ was purchased from Sigma Aldrich^®^ (Bangalore, India). Polysorbate 80 and Triton X 100^®^ were purchased from Himedia^®^ (Mumbai, India). All other chemicals used were of analytical grade.

### Nanoparticles synthesis

**Figure 1.**
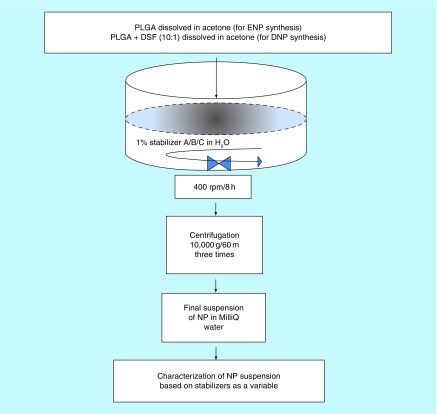
**Schematic representation of disulfiram-loaded poly-lactide-co-glycolide nanoparticles synthesis.** Disulfiram-loaded PLGA nanoparticles and empty nanoparticles were synthesized using three different compounds as stabilizers, namely, Polsorbate 80, Triton X-100^®^ and Pluronic 188^®^; in 1% concentration in 30 ml of Milli-Q^®^ water. Thus, for each of the three stabilizers, two distinct type of NPs were produced, a Disulfiram loaded and an empty PLGA NP, thus resulting in total of six types of NPs. NP: Nanoparticle; PLGA: Poly-lactide-co-glycolide.

Nanoparticles were synthesized by nanoprecipitation method, as described by Fessi et al. [9]. In brief, 50 mg of PLGA was dissolved in 5 ml of acetone. In a separate batch, 50 mg of PLGA was dissolved in 5 ml acetone together with 5 mg of DSF. Solutions were then dispersed in 30 ml of each 1% PS80, T100 and P188. Resulting colloidal solutions of nanoparticles were magnetically stirred for 8 h at room temperature (RT), in order to remove redundant acetone. The colloidal solutions of NPs were finally centrifuged at 10,000 × *g* (Eppendorf^®^, R5410, Hamberg, Germany) three-times to remove the residual stabilizers and free DSF that was present in the colloidal solution. The final pellet of nanoparticles is suspended in Milli-Q^®^ water and stored in 4°C (Figure 1). Overall, six differentially stabilized nanoparticles were synthesized in three batches; each batch representing one of the three stabilizers used in other words, PS80, T100 and P188. Further, each batch had two types of NPs, an empty NP and a DSF-loaded NP. The morphologies and size of the nanoparticles were then studied by scanning electron microscopy (S-3400N, Hitachi^®^, Japan), and dynamic light scattering technique (ZetaSizer^®^, Malvern Instruments^®^, UK), as previously reported [5].

### Differential scanning calorimetry

The glass transition temperatures (Tg) of the differentially-stabilized NPs were studied using differential scanning calorimeter (TA Instruments^®^, DSC Q20, USA). Known weight (10 mg) of NPs were kept in aluminium pan and thus sealed by hydraulic press. The heat flow was measured as the change in weight of the aluminium pan, thus reporting heat flow as Watt per gram sample as the temperature rises from 25 to 100°C. The final data were analyzed using Origin^®^ software.

### X-ray diffraction analysis

Crystallinity studies of all the six types of NPs were done by x-ray diffractometer (Rigaku^®^ Ultima IV, Tokyo, Japan). The system was configured to scanning range (2-theta) of 10–40° and a scanning speed of 0.02°/min. X-ray of a wavelength 1.54Å was bombarded from Cu-Kα x-ray generator. The data were finally analyzed by Origin^®^ software.

### Raman spectrum analysis

Drug–polymer interactions in NPs were studied by Raman spectral analysis using Renishaw^®^ inVia Raman microscope, RE 04 (Gloucestershire, UK). NPs and the physical mixture of the components of NPs were scanned for 500–2000 cm^-1^, using 785 nm diode laser. The laser power was set up at 5 mW, and the exposure time was 20 s. The peak analysis was done by Wire 3.14 software, and the graphs were plotted using Origin^®^ software.

### Isothermal titration calorimetry

Thermodynamics study of drug–polymer interaction and nanoparticles synthesis were studied by isothermal titration calorimetry (TA Instruments^®^, LV NanoITC^®^, USA). In drug–polymer interaction study, titrant taken in the syringe was DSF (10 μM), while the cell contained PLGA (30 μM). In subsequent experiments, 1% stabilizers were mixed with 30 μM PLGA.

In nanoparticles synthesis study, a solution containing mixture of 10 μM DSF and 30 μM PLGA was injected from the syringe into Milli-Q^®^ water containing 1% stabilizers, in different combinations. Briefly, 50 μl of titrant was taken in syringe, while cell volume was 170 μl. the system was calibrated using 40 mM Tris and 1 mM HCl and further configured for 25 injections, with 2 μl being injected each time. The syringe rotation was kept at 150 rpm. Time interval between successive injections was 300 s. The final data were analyzed by NanoITC Analyze^®^ software, using independent model.

### *In silico* docking

*In silico* interaction study of DSF and PLGA with stabilizers P188, T100 and PS80 was done by Autodock 4.0 software [10]. The structures were obtained from Pubchem database in 3D SDF format and were converted into PDB format using open babel software (Table 1). The ligands were treated with gastrier charges for the interaction studies. The binding energies and H-bond formation was considered as the criteria for the interaction study.

**Table 1.** **Pubchem chemical ID of molecules used for docking by AutoDock 4.0.**

**Molecule**	**PubChem CID**	**Molecule**	**Pubchem CID**
Polysorbate 80	86289060	Disulfiram	3117

Triton X-100	5590	PLGA	23111554

Pluronic 188	24751		

PLGA: Poly-lactide-co-glycolide.

## Results

### Nanoparticle synthesis

**Figure 2.**
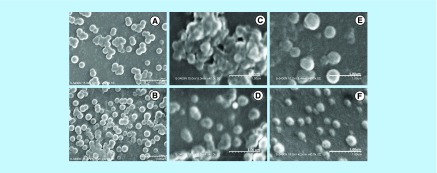
**Morphology of disulfiram-loaded, and empty poly-lactide-co-glycolide nanoparticles using scanning electron microscopy technique.** SEM images of **(A)** polysorbate 80-stabilized empty PLGA nanoparticles; **(B)** polysorbate 80-stabilized disulfiram-loaded PLGA nanoparticles; **(C)** pluronic 188^®^-stabilized empty PLGA nanoparticles; **(D)** pluronic 188^®^-stabilized Disulfiram-loaded PLGA nanoparticles; **(E)** Triton X-100^®^-stabilized empty PLGA nanoparticles; **(F)** Triton X-100^®^-stabilized disulfiram-loaded PLGA nanoparticles. PLGA: Poly-lactide-co-glycolide; SEM: Standard error of the mean. **(B)** Reproduced with permission from [5].

Overall, two batches of three types of nanoparticles were produced. The first batch included three types of empty nanoparticles (ENPs) with the three individual stabilizers in other words PS80, T100 and P188, while the second batch included DSF-loaded nanoparticles (DNPs) with the same stabilizers as used to produce ENPs. The morphologies of all the nanoparticles were observed to be near-spherical (Figure 2). The mean diameter size of all the nanoparticles was reported to be ranging between 100 and 125 nm (data not included).

### Differential scanning calorimetry

**Figure 3.**
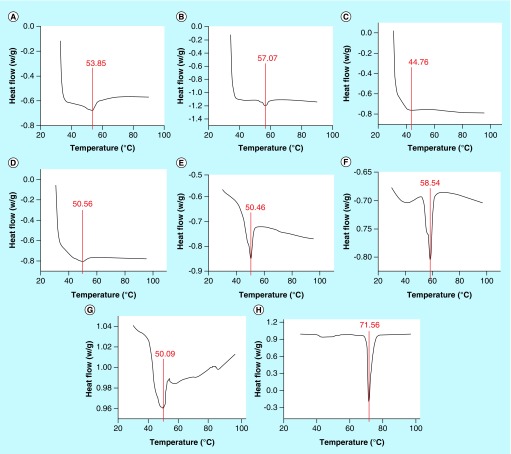
Glass temperature change in disulfiram-loaded poly-lactide-co-glycolide nanoparticles using different stabilizers*. Differential scanning calorimetry graph plots of heat flow (W/g) against temperature ranging from 25 to 100°C of **(A)** polysorbate 80-stabilized, disulfiram-loaded nanoparticles; **(B)** polysorbate 80-stabilized empty nanoparticles **(C)** Triton X-100^®^-stabilized, DSF-loaded nanoparticles and **(D)** Triton X-100^®^-stabilized empty nanoparticles; **(E)** pluronic 188^®^-stabilized DSF-loaded nanoparticles and **(F)** pluronic 188^®^-stabilized empty nanoparticles; **(G)** PLGA polymer alone and **(H)** PLGA polymer-disulfiram powder physical mixture. **(A, C & E)** have been reprinted with permission from [5] © Future Science Group^®^. DSF: Disulfiram; PLGA: Poly-lactide-co-glycolide.

Differential mechanical strengths of the NPs were evident from differential scanning calorimetry. Equivalent amount of NPs showed significantly different Tg values, although the amount of heat flow were observed to be similar. The Tg of T100-stabilised DSF-loaded NPs (T100 DNPs) was noted to be lowest, followed by P188-stabilized DSF-loaded NPs (P188 DNPs), while PS80-stabilized DSF-loaded NPs (PS80 DNPs) were observed to show highest Tg, though significantly near to Tg of P188 DNPs (Figure 3). However, in all the three NPs, the heat flow change remained consistent between -0.6 and -0.8 W/g. Tg of PLGA was 50.9°C, while that of physical mixture of PLGA and DSF was recorded to be 71.56°C. ENPs based on all three stabilizers were observed to show higher Tg than their DSF-loaded counterpart, in other words, DNPs. Tg of all the nanoparticles varied from that of plain PLGA. Tg of PS80 NPs and P188 NPs significantly elevated while for T100 NPs, Tg significantly reduced, as compared with PLGA (Figure 3).

### X-ray diffraction studies

**Figure 4.**
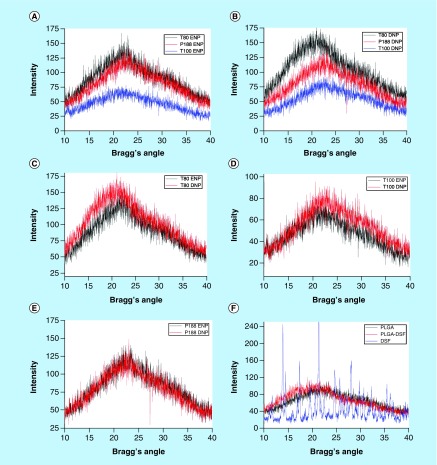
**Change in degree of crystallinity of poly-lactide-co-glycolide nanoparticles under the influence of various stabilizers.** Comparative X-ray diffraction graph plots of intensity peaks against Bragg's angle (2-theta) ranging from 10 to 45 degrees of **(A)** Polysorbate 80-, Triton X-100^®^- and Pluronic 188^®^-stabilized empty PLGA nanoparticles; **(B)** polysorbate 80^®^-, Triton X-100^®^-, and Pluronic 188^®^-stabilized disulfiram-loaded PLGA nanoparticles; **(C)** polysorbate 80-stabilized empty and disulfiram-loaded PLGA nanoparticles; **(D)** Triton X 100^®^- stabilized empty and disulfiram-loaded PLGA nanoparticles; **(E)** Pluronic 188^®^- stabilized empty and disulfiram-loaded PLGA nanoparticles; **(F)** PLGA polymer, Disulfiram and physical mixture of PLGA and disulfiram. DNP: Disulfiram nanoparticle; ENP: Empty nanoparticle; PLGA: Poly-lactide-co-glycolide.

X-ray powder diffraction results obtained in PLGA did not show any characteristic peak at any of the Bragg's angles. However, a thread-line of the graph indicated that PLGA show a single broad peak between 10 and 40° Bragg's angles (Figure 4). Contrary to that, free DSF showed multiple characteristic peaks of very high intensity between 10 and 40° Bragg's angles. Physical mixture of free DSF and PLGA showed dominance of PLGA in the graph pattern, though the thread-line was observed to shift toward left, having greater intensity, followed by rapid decline of the thread-line, below PLGA. X-ray powder diffraction of NPs showed that there were absolutely no peaks in any of the nanoparticles. The graph plots were very much the same as plain PLGA. However, on closer observation, the thread lines showed that intensities and broad peak of each of the nanoparticles were different. Both the PS80 NPs (DNP & ENP) had maximum intensity and sharpest peak, followed by P188 and finally T100. However, the comparison between ENPs and DNPs showed that intensity gap between PS80 ENP & P188 ENP was much less than their DNP counterpart.

### Raman spectral analysis

**Figure 5.**
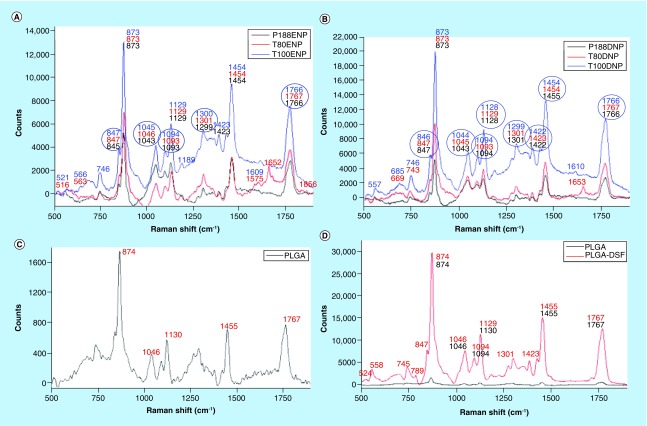

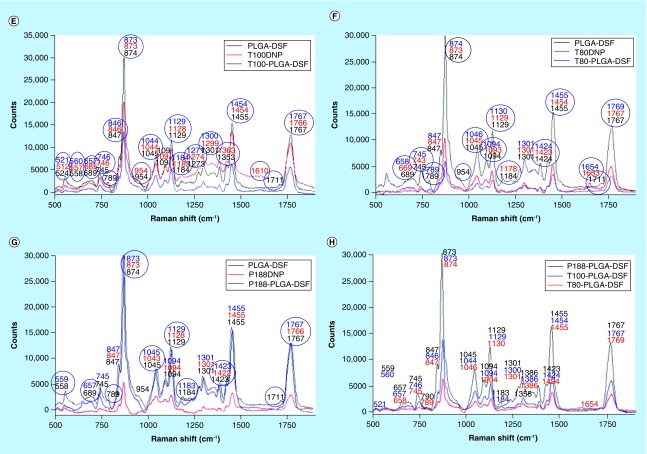
**Raman peak shift of functional groups present in poly-lactide-co-glycolide nanoparticles using various stabilizers.** Comparative Raman spectrum graph plots ranging from 500 to 1875 cm^-1^ of **(A)** polysorbate 80-, Triton X-100^®^-, and Pluronic 188^®^-stabilized empty PLGA nanoparticles; **(B)** polysorbate 80-, Triton X-100^®^- and Pluronic 188^®^-stabilized disulfiram-loaded PLGA nanoparticles; **(C)** PLGA polymer and **(D)** PLGA polymer and physical mixture of PLGA and disulfiram powder; **(E)** Triton X-100^®^- stabilized nanoparticles and physical mixture of PLGA-disulfiram and PLGA-disulfiram-Triton X-100^®^; **(F)** polysorbate 80-stabilized nanoparticles and physical mixture of PLGA-disulfiram and PLGA-disulfiram-polysorbate 80; **(G)** Pluronic 188^®^- stabilized nanoparticles and physical mixture of PLGA-disulfiram and PLGA-disulfiram-Pluronic 188; **(H)** physical mixture of individual stabilizers-PLGA-disulfiram. DNP: Disulfiram nanoparticle; DSF: Disulfiram; ENP: Empty nanoparticle; PLGA: Poly-lactide-co-glycolide.

Raman peak analysis was done by Renishaw Wire 1.43^®^ software, whereas overlapping spectrums of different combinations of NPs were plotted by Origin^®^ software. Numbers of lateral peak shifts in NPs (both ENPs & DNPs) were less as compared with the physical mixture of the constituents of ENPs and DNPs (Figure 5). While comparing the number of lateral peak shifts between empty and drug-loaded NPs, drug-loaded NPs seemed to have more number of lateral shifts among their respective stabilizers. Among individual stabilizers and physical mixture of their NPs, T100 showed maximum number of lateral peak shifts, followed by PS80, while P188 showed minimum number of lateral peak shifts (Figure 5).

### Isothermal titration calorimetry

The drug–polymer interaction in acetone medium was studied in presence and in absence of 1% of the three stabilizers. In absence of stabilizers, interaction between DSF and PLGA had the highest association constant, as compared with DSF-DSF and PLGA-PLGA interaction (Table 2). Stoichiometry of DSF–PLGA interaction was also noted to be highest when compared with DSF-DSF and PLGA–PLGA interaction. Change in entropy was greater than change in enthalpy, among all the three combinations. Though, the difference between change in enthalpy and entropy was much lesser in PLGA-PLGA interaction when compared with DSF-DSF and DSF–PLGA interactions. DSF–PLGA interaction in presence of stabilizers showed significantly varying results as compared with the DSF–PLGA interaction in absence of stabilizers. P188 showed minimum dissociation constant (Kd), followed by T100 and finally PS80. However, DSF–PLGA interaction, in absence of stabilizers showed minimum Kd. Stoichiometry of DSF–PLGA interaction in the presence of P188 was also noted to be highest; however, stoichiometry of PS80 was observed to be greater than T100, as contrary to the Kd (Table 2).

**Table 2.** **Isothermal titration calorimetry data of drug–polymer interaction in presence of various stabilizers.**

		**Ka (1/M)**	**Kd (M)**	**n**	**ΔH (kJ/Mol)**	**ΔS (J/Mol.K)**
Drug–polymer interaction in absence of stabilizer	Acetone to acetone	3.915e + 01	2.554e - 02	0.024	0.046	30.65

	DSF to DSF in acetone	1.000e + 10	1.000e - 10	0.289	1999	6899

	DSF to PLGA in acetone	7.232e + 09	1.383e - 10	286.2	1999	6896

	PLGA to PLGA in acetone	1.855e + 09	5.391e - 10	138.6	1999	6893

Drug–polymer interaction in presence of stabilizer	DSF to PLGA in 1% P188 acetone	4.792e + 09	2.087e - 10	316.9	150.9	683.4

	DSF to PLGA in 1% T100 acetone	2.294e + 09	4.358e - 10	2.685	-37.49	53.44

	DSF to PLGA in 1% PS80 acetone	9.999e + 08	1.000e - 09	31.89	-20.13	104.8

ΔH: Change in enthalpy; ΔS: Change in entropy; DSF: Disulfiram; Ka: Association constant; Kd: Dissociation constant; kJ: Kilo joule; Mol.K: Mole. Kelvin; n: Stoichiometry; PLGA: Poly-lactide-co-glycolide.

ITC experiments were also performed for both ENPs and DNPs synthesis. While entropy change (ΔS) among both types of NP synthesis was found to be of the order of P188 > T100 > PS80, enthalpy change (ΔH) among ENPs were observed to be greater than ΔH of DNPs. Stoichiometry of ENPs were in the order of PS80 > T100 > P188, while that of DNPs were observed to be reverse of ENPs. Kd of both ENPs and DNPs were observed to be of the order PS80 > T100 > P188 (Table 3).

**Table 3.** **Isothermal titration calorimetry data of nanoparticles synthesis.**

		**Ka (1/M)**	**Kd (M)**	**n**	**ΔH (kJ/Mol)**	**ΔS (J/Mol.K)**
PLGA in acetone titrated with 1% stabilizers in water	PLGA to 1% P188 water	1.124e + 09	8.895e - 10	194.7	-0.143	172.8

	PLGA to 1% TX100 water	2.389e + 07	4.187e -08	203.8	-0.063	141

	PLGA to 1% PS80 water	5.353e + 03	1.868e -04	408.8	-0.029	71.28

PLGA + DSF in acetone titrated with 1% stabilizer in water	DSF + PLGA to 1% P188 water	1.676e + 09	5.967e -10	304.7	-0.192	175.9

	DSF + PLGA to 1% T100 water	2.522e + 09	3.965e -10	98.59	-0.080	179.7

	DSF + PLGA to 1% PS80 water	1.000e + 01	1.000e -01	18.31	-0.660	16.93

DSF: Disulfiram; PLGA: Poly-lactide-co-glycolide.

### *In silico* docking studies

**Figure 6.**
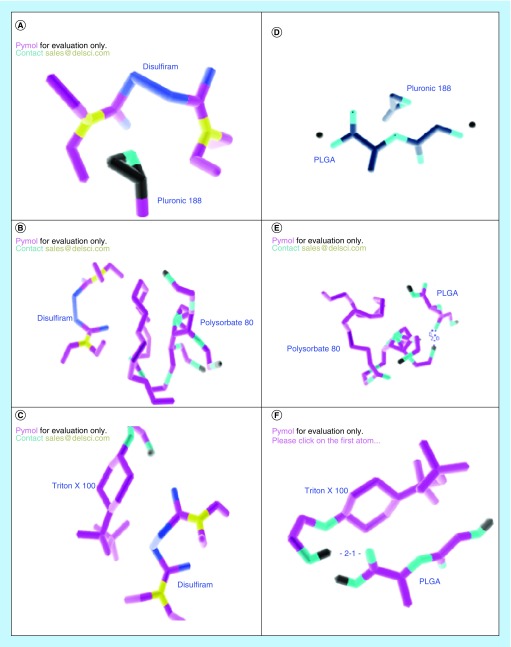
**Intermolecular interaction of various monomers and disulfiram present in poly-lactide-co-glycolide nanoparticles.** *In silico* molecular docking using Auto Dock 4.2 was performed between **(A)** Disulfiram and monomer of Pluronic 188^®^ **(B)** Disulfiram and polysorbate 80 **(C)** Disulfiram and Triton X-100^®^ **(D)** monomers of PLGA and Pluronic 188^®^ **(E)** monomers of PLGA and polysorbate 80 **(F)** monomers of PLGA and Triton X-100^®^. PLGA: Poly-lactide-co-glycolide.

The interactions of DSF and PLGA with P188 showed minimum free energies compared with the T100, whereas PS80 was observed to show maximum free energy. While all other stabilizers showed some level of H-bond formation with PLGA, P188 did not show any H-bond interaction (Figure 6). Free energy, as a result of DSF–PLGA interaction, was observed to be lowest as compared with any DSF-stabilizer or PLGA–stabilizer interaction (Table 4).

**Table 4.** **Molecular docking studies of components of nanoparticles.**

**Stabilzer**	**Disulfiram–stabilizer interaction**		**PLGA–stabilizer interaction**	

	**Free energy change**	**H-bond formation**	**Free energy change**	**H-bond formation**
Polysorbate 80	6.41	1	5.18	1

Triton X-100	-0.22	2	-0.6	2

Pluronic 188	-0.49	nil	-0.73	Nil

PLGA: Poly-lactide-co-glycolide.

## Discussion

Stabilizers have not been given due attention in polymeric nanoparticles research. They are an integral component of polymeric nanoparticles and play a critical role in drug–polymer interaction, apart from surface modification. Stabilizers directly influence the physicochemical properties of NPs such as drug-releasing profile, drug entrapment efficiency, crystallinity, hyrdophobicity, agglomeration capabilities, BRT, etc. Degradation and drug release kinetics may be precisely controlled by manipulating the physicochemical properties of the NPs, such as molecular weight of polymer, polydispersity index, hydrophobicity and crystallinity [11].

Tg refers to the temperature at which the molecules transit from glass-like property to fluid-like property. Such a phenomenon is known as plasticization [12]. Tg reflects the mechanical strength of the NPs. The varying Tg values and their differences in heat flow during transitions reflect the fact that the stabilizers significantly influence the physical properties of the PLGA NPs. It provides an insight on how the stabilizers may influence the mechanical strength of entire matrix of NPs and not just the surface, thus behaving more like an emulsifier rather than just a surfactant. A similar insight into the stabilizers working as cross-linking agents has previously been reported [13]. Therefore, an argument between the terminologies of surfactant and emulsifier could be debated. However in polymeric nanotechnology, it is more or less interchangeably used. The P188 DNPs showed a sharp reverse peak region in the Tg, thus indicating that P188 DNPs melt rapidly, but the PS80 DNPs showed a much broader reverse peak region in the Tg, indicating a more durable nature of PS80 DNPs as compared with P188 DNPs. Since, Tg of PS80 and P188 NPs are observed to be above Tg of PLGA polymer and that of T100 NPs was below Tg of PLGA polymer, P188 and PS80 stabilizers were observed to show antiplasticizing effect, while T100 tend to show plasticizing effect on PLGA. Plasticizing effect of T100 in PLGA NPs corroborated with a previous report on PLGA microparticles [14].

Rigidity of NPs is one of the important physicochemical parameters that influence BRT of NPs. It has been reported that high Tg NPs have significantly higher BRT as compared with low Tg NPs [15]. On the other hand, it is also reported that low Tg NPs provide greater flexibility and elevated level of surface interactions between tissues and NPs [16]. Therefore, it may be that among all three DNPs, PS80 DNPs could have the highest BRT, thus being able to have better passive targeting capability. This observation is in consistency with the fact that polysorbate 80 is a known surface modifier that increases BRT. It may also be noted that T100 DNPs could show maximum surface interaction, thus having maximum tissue penetration and cellular uptake. Further, all of DNPs were observed to have lower Tg than their empty NP counterpart, while Tg of DSF and physical mixture of DSF and PLGA individually is much higher (76°C) than all of NPs and polymers, thus suggesting that DSF has plasticizing effect on NPs. It may also be said that DNPs may get entrapped into the tissue easily as compared with their ENP counterparts. Further, all of the NPs were observed to show more or less single reverse Tg peaks, thus indicating that all components of a given NP were highly miscible with each other at molecular level, thus imparting a single amorphous state, however the degree of miscibility may vary as is evident from varying enthalpies [17].

**Figure 7.**
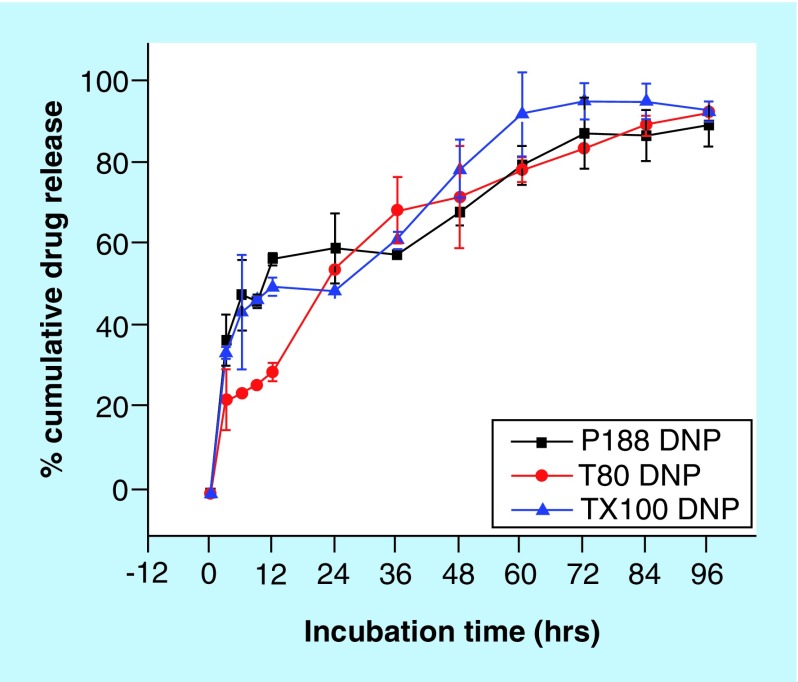
***In vitro* release study of the disulfiram-loaded poly-lactide-co-glycolide nanoparticles using various stabilizers [7].** The drug released from disulfiram-loaded nanoparticles was observed at regular time interval of 3 h for the first 12 h and subsequently at 12 h interval for next 84 h. Thus, a total of 96 h *in vitro* release study was performed independently for three experiments, and statistical analysis was done. DNP: Disulfiram nanoparticle. Reprinted with permission from [5] © Future Science Group.

**Figure 8.**
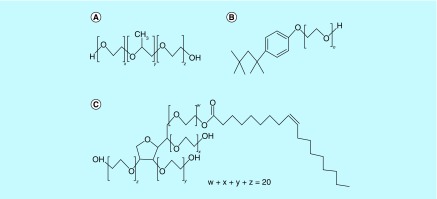
**Empirical chemical structures of the stabilizers under study.** Chemical structures of **(A)** Pluronic 188^®^ **(B)** Triton-X 100^®^ **(C)** Polysorbate 80.

**Figure 9.**
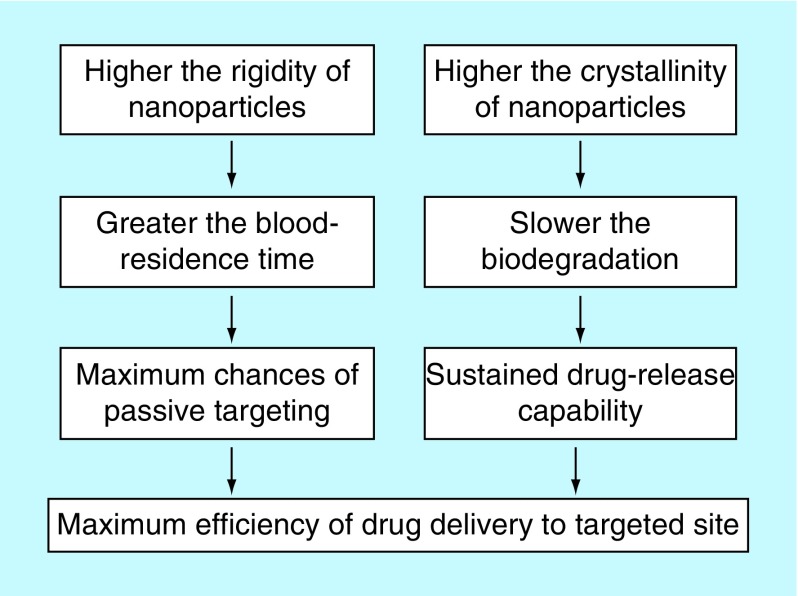
**Influence of rigidity and crystallinity on physicochemical properties of poly-lactide-co-glycolide nanoparticles.** Schematic representation of the effect of stabilizers on physicochemical properties of PLGA NPs, which in turn affect the drug-releasing capability of PLGA NPs. NP: Nanoparticle; PLGA: Poly-lactide-co-glycolide.

Crystallinity is another critical parameter which markedly affects the physicochemical behavior of NPs, such as mechanical strength, water-adsorbing capability, biodegradation, drug-releasing kinetics, etc. [18]. Previously, there has been conflicting reports about the influence of crystallinity on biodegradation of polymers such as poly-lactic acid. While some reports suggest that increase in crystallinity may result in increase in biodegradation, there are others which suggest that rate of biodegradation decreases [19,20]. In our case of differentially stabilized PLGA NPs, it was observed that T100 NPs showed minimum crystallinity, P188 NPs showed higher crystallinity than T100 NPs, while PS80 NPs showed the highest crystallinity. Thus suggesting that rate of biodegradation (increasing or decreasing) would be in order of PS80 > P188 > T100. To understand whether the rate of biodegradation of NPs increases or decreases in the our case of differentially stabilized NPs, we studied the drug-releasing kinetics of these NPs that was previously reported (Figure 7) [5]. T100 NPs released all its drug-load in shortest time (60 h), followed by P188 NPs (72 h), while PS80 NPs showed sustained drug-release for the longest time period (96 h), thus suggesting that T100 NPs degraded fastest so as to be able to release its entire drug-load, followed by P188 NPs, while PS80 NPs underwent slowest degradation, thus, being able to release the drug for longest time period. Therefore, from these observations, we may infer that rate of biodegradation decreases with increase in crystallinity in case of DSF-loaded PLGA NPs. This could further be inferred from the fact that PS80 has long hydrocarbon chains which makes it less prone to hydrolysis when compared with the other two stabilizers which are more prone to hydrolysis due to the presence of extensive –C-O-C- bonds (Figure 8). Hence, stabilizers significantly influence the rigidity and crystallinity of the nanoparticles, which in turn affects the passive-targeting, drug localization and drug-releasing properties of PLGA NPs (Figure 9).

Raman spectral analysis is an apt technique which gives an insight of real-time drug-polymer and polymer–stabilizer interaction. NPs of different stabilizers were compared with their physical mixture counterparts. Similar spectrum was observed in all the NPs when compared with their physical mixture counterpart, thus suggesting that no new covalent bonds were formed between the drug, polymer and stabilizers. However, significant numbers of lateral peak shifts were observed. Among all peak shifts that were observed, peak shift in NPs were always toward the lower wave number, as compared with the physical mixture counterparts. One of the reports suggests that peak shifts toward lower wave number is mainly due to H-bonding formation [21]. This could be true because, most of the peak shifts observed were related to characteristic functional groups that could potentially be involved in H-bond formation. Additionally, *in silico* docking studies also showed the highest H-bond formation in T100, followed by PS80, while P188 did not show any H-bond formation (Table 4), thus, conforming with Raman peak shift results. However, we do not rule out any presence of H-bond formation in P188 NPs, due to the presence of functional groups that could potentially be involved in H-bond formation. Certain other characteristic groups that could be involved in hydrophobic interactions, such as aliphatic and aromatic side chains were also observed to undergo lateral peak shifts, thus, suggesting differential hydrophobic interactions in different NPs. This could precisely be the reason for imparting specific physicochemical properties to NPs.

Thermodynamic parameters such as binding constants, stoichiometry, and enthalpy and ΔS are of immense importance in order to understand drug–polymer interaction. A drug–polymer interaction generally involves various types of nonbonded interactions such as electrostatic, H-bonding, dipole-dipole, Vander-Waal and hydrophobic interactions, precisely depending on the kind of functional groups present. Considering the functional groups involved in the polymer, stabilizers and drug under study, it clearly suggests three major types of interactions that are possible, viz a viz, H-bonding, Vander-Waals and hydrophobic interactions. Some reports suggest that Vander-Waal and hydrophobic interactions are determined by enthalpy and entropy changes of the system. Vander-Waal interactions are enthalpy-driven (ΔH > TΔS) [22] whereas, hydrophobic interactions are entropy-driven (ΔH < TΔS) [23]. Our data clearly shows that all of the interactions (in presence and absence of stabilizers) are entropy-driven, thus, suggesting that the drug-polymer/drug-stabilizer interactions are dominantly hydrophobic in nature, while not ruling out H-bonding as predicted from Raman spectral analysis.

Highest ΔS during drug–polymer interaction was observed in absence of stabilizer, followed by DSF-PLGA interaction during NP synthesis in presence of P188, T100 and, PS80 respectively. Entropy is directly related to the degree of freedom of the molecules in interaction [24]. Maximum randomness is observed in drug–polymer interaction in which there are no stabilizers involved, while PS80 provides minimum internal degrees of freedom as compared with T100. P188 provides highest internal degrees of freedom as compared with the other two stabilizers. This may be attributed to the hydrophobic entity present in each of these stabilizers. While in P188, there are long chains of polyethylene and polypropylene oxide entities, PS80 has a long aliphatic chain of oleic acid, and T100 has a branched aliphatic chain and an aromatic ring in its structure (Figure 8). This suggests that with increasing degree of hydrophobic interaction, entropy decreases, due to decrease in free movement of molecules entrapped in the polymer, just as in case of cyclodextrans-chlorophenols interactions, due to H-bonding [25].

Kd is a parameter that determines the binding affinity. Smaller the Kd, greater is the binding affinity among the constituents of NPs. Among individual stabilizers, the Kd of ENPs is observed to be lower than Kd of DNPs. This could be due to the interference in hydrophobic interactions, or due to the presence of disulfide, thiocarbonyl and imines in DSF. This assumption is in concurrence with DSC results, where Tg of NPs is lowered in all the stabilizers due to the presence of DSF.

P188 and T100 NPs may have strong intermolecular interactions as compared with PS80 NPs. However, it is observed that P188 and T100 NPs are more prone to biodegradation by hydrolysis, thus resulting in release of drug in bursts, as observed during *in vitro* release study (Figure 7). PS80 NPs, due to extensive aliphatic/hydrophobic chains, are comparatively less prone to hydrolysis, thus releasing the drug due to consistent weakening of stabilizer-polymer interaction, minimally affected by hydrolysis. Lowering of Kd suggests that P188, followed by T100, strongly binds the drug and the polymer, generating high binding affinity, while PS80 lowers the binding affinity significantly. *In silico* docking studies further corroborate with Kd data obtained in ITC (Table 2). However, biodegradation may not be necessarily influenced by binding affinities of drug–polymer interaction, since biodegradation is known to be majorly due to hydrolysis [26]. Higher the hydrophobicity, greater would be the water repelling capability, thus lesser would be the chance for undergoing hydrolysis and in turn, slower would be the biodegradation. In other words, drug release may be dominatingly influenced by the rate of biodegradation than the binding affinity. A second plausible inference could be derived from our *in vitro* release study (Figure 7) [5] and binding affinity data. Minimum drug–polymer interaction in PS80 may be the reason for a uniform drug release pattern. In P188 and T100 NPs, the peripheral drug may be released in bursts because of aqueous corrosion, however, the centrally-entrapped DSF could be possibly held into the NP matrix due to limited aqueous exposure that prevents degradation of polymer and stabilizers, along with strong binding affinity as observed in P188 and T100 NPs.

Stoichiometry of drug–polymer interaction in presence of individual stabilizers were observed to be in order of P188 > PS80 > T100. This may be attributed to the molecular weight of individual stabilizers. Since, equivalent amount of stabilizers were used in all three experimental setups, in other words 1%, molecular weights of all the three stabilizers vary significantly. P188 has the highest molecular weight (7500 g/mol), followed by PS80 (1310 g/mol) and T100 (638 g/mol). Owing to the polymeric nature of P188, stoichiometry of P188 was found to be highest in other words, more number of P188 monomers interacted with DSF, followed by PS80, while T100 showed least stoichiometry. During nanoparticles synthesis, stoichiometry of DNPs was observed to be reverse of ENPs. In ENPs, number of molecules of each of the stabilizers that interact with PLGA is in the order of PS80 > T100 > P188. This could probably be because of dominating hydrophobic side chains in PS80, followed by T100 and last P188. However for DNPs, it was observed to be in reverse order. This may be attributed to increasing number of DSF being entrapped inside PLGA-Stabilizer emulsion, which is evident from the data of drug-entrapment efficiency, thus competing with stabilizers to interact with PLGA.

Nanoparticles synthesis is observed to be essentially entropy-driven. However, it is also enthalpy-favored spontaneous interaction because the ΔH is -ve in all cases of NP synthesis. It further suggests that major type of interaction among NP constituents is hydrophobic type, though it does not rule out existence of other types of interactions such as Vander-Waal, electrostatic and H-bonding. Among all the three stabilizers, P188 is observed to show most negative enthalpy and maximum entropy, followed by T100, and PS80. Therefore, from these observations, we infer that drug–polymer interaction may be significantly influenced by the kind of stabilizers used, yet biodegradation, drug-release capabilities and external environment interactions may or may not completely be dependent drug–polymer interaction because of many other factors that come into play, such as enthalpy and entropy of the NP system, and NP interactions with external environment.

## Conclusion

Stabilizers influence critical physicochemical parameters such as glass transition, crystallinity and drug–polymer interaction. This may lead to imparting various objective-oriented characteristics to a specific NP, in a way, streamlining the passive-targeting concept of NP as a drug-delivery vehicle. Further, we also conclude that the hydrophobic entities of stabilizers are critical for developing a DSF-PLGA NP. Extensive polyethylene glycol residues help in generating a rigid NP; however, hydrophobic entities of the stabilizers are keys to various other physicochemical characteristics of NPs. This further expands the domains of research in terms of drug–polymer interaction, and the mechanics involved in it. Future research may involve several other amphiphilic molecules as stabilizers and extensive work may be done on optimizations of specific drug–polymer interactions based on specific physicochemical characteristics in consideration.

## Future perspective

Tailor-made nanoparticles hold key to successful implementation of nanotechnology in drug-delivery sciences. There is a lot of scope for improvisation of nanoparticles as drug-delivery vehicle. The current study is one among many efforts to identify the right combinations of polymer, drug and stabilizer in order to improvise drug-loading and release capability of nanoparticles. Extended studies on comparative drug-release patterns using a hydrophilic and a hydrophobic entity as model drugs may be performed in immediate future in order to further substantiate our current research perspective. Extensive work based on various other polymers and drugs may also be studied in order to understand drug–polymer interactions and their implications in drug-delivering nanoparticles. At the same time, application of raman spectroscopy, ITC and molecular docking studies needs to be further substantiated by performing similar experiments based on various other types of polymers, drug and stabilizers.

**Executive summary**

- Stabilizers play critical role in physicochemical characterization of biodegradable poly-lactide-co-glycolide (PLGA) nanoparticles.
- Polysorbate 80-stabilized PLGA nanoparticles were observed to be most rigid, while Triton X-100-stabilized PLGA nanoparticles was observed to be most crystalline in physical property.
- All empty nanoparticles were observed to be less rigid and less crystalline as compared with disulfiram-loaded counterparts.
- Among the three stabilizers studied, polysorbate 80 was observed to be most compatible as a stabilizer against disulfiram-PLGA combination.
- Isothermal calorimetry, Raman spectroscopy and *in silico* docking studies could be a reliable tool to predict and identify potential biodegradable polymers and their compatible stabilizers.
- Compatible drug–polymer interaction could also be predicted using the above mentioned characterization tools.
